# The Relationship Between Perceived Organizational Support, Perceived Professional Benefits, and Professional Values Among Nurses: Variable‐Centered and Individual‐Centered Analyses

**DOI:** 10.1155/jonm/9608844

**Published:** 2026-04-29

**Authors:** Wu Zhengyu, Sun Lijuan, Yang Haibo, Zhang Yani, Meng Hui, Zhang Zhongxia, Yin Xiaodan, Bai Yunxia, Cao Jianqin

**Affiliations:** ^1^ Key Laboratory of Basic Research and Health Management on Chronic Diseases in Heilongjiang Province, Harbin Medical University, Daqing Campus, Daqing, Heilongjiang, China, hrbmu.edu.cn; ^2^ Department of Nursing, Daqing Third Hospital, Daqing, Heilongjiang, China; ^3^ Department of Geriatrics, Daqing Third Hospital, Daqing, Heilongjiang, China; ^4^ Department of Traditional Chinese Medicine, Daqing Third Hospital, Daqing, Heilongjiang, China; ^5^ Outpatient Department, Daqing Third Hospital, Daqing, Heilongjiang, China; ^6^ Department of Sleep Medicine, Daqing Third Hospital, Daqing, Heilongjiang, China

**Keywords:** latent profile analysis, nurses, perceived organizational support, perceived professional benefits, professional values

## Abstract

**Objective:**

To examine the relationships among perceived organizational support, perceived professional benefits, and professional values in nurses.

**Methods:**

A cross‐sectional survey was conducted with 742 nurses from five hospitals in China. Participants completed measures of perceived organizational support, perceived professional benefits, and professional values. Mediation analysis and latent profile analysis were employed.

**Results:**

Perceived professional benefits partially mediated the relationship between perceived organizational support and professional values (indirect effect = 0.327, 95% CI [0.249, 0.414]). Five distinct profiles of perceived organizational support and perceived professional benefits were identified, which differed significantly in professional values (*F* = 171.282, *p* < 0.001).

**Conclusions:**

Perceived organizational support influences professional values both directly and indirectly through perceived professional benefits. The heterogeneity in nurses’ perceptions underscores the need for targeted interventions tailored to different subgroups.

## 1. Introduction

Nurses’ professional values are the behavioral norms recognized by nursing professionals, which are internalized through training and learning, influencing and guiding individual behavior and job satisfaction, and are the cornerstone of nursing practice and improving the quality of nursing services [1]. The formation of professional values is a durative and long‐term process. Existing literature indicates that the correctness of values directly affects nurses’ occupational mental health. Only under the guidance of positive nurses’ professional values can nursing personnel generate a sense of identification with their profession, thereby enhancing their core work capabilities to provide safer, higher quality, and more efficient services for patients [2].

The importance of professional values in nursing cannot be overstated. However, the nursing profession globally faces significant challenges, with turnover rates among nurses ranging from 15% to 44% worldwide [3, 4]. Despite the large size of China’s nursing workforce, staffing levels remain insufficient, and turnover rates continue to be high [5]. These statistics underscore the urgent need to understand factors that strengthen nurses’ professional values and, consequently, their professional commitment and retention.

Perceived organizational support (POS), defined as employees’ global beliefs concerning the extent to which the organization values their contributions and cares about their well‐being, was first conceptualized by Eisenberger and colleagues [6]. According to organizational support theory (OST), employees personify the organization, perceiving favorable or unfavorable treatment as an indication that the organization favors or disfavors them [6, 7]. This personification leads employees to reciprocate POS with increased commitment, effort, and positive attitudes toward the organization.

OST is fundamentally grounded in social exchange theory, which posits that social relationships are governed by norms of reciprocity—individuals who receive benefits feel obligated to return benefits to the benefactor [8]. Within the workplace context, when nurses perceive that their organization values their contributions and cares about their well‐being, they experience a sense of indebtedness that motivates them to reciprocate through positive work attitudes and behaviors. This reciprocal process operates through two primary mechanisms. First, POS fulfills socioemotional needs, leading nurses to incorporate organizational membership into their social identity and develop stronger affective commitment. Second, POS strengthens nurses’ beliefs that the organization recognizes and rewards increased effort, enhancing their expectancy that hard work will be valued [7].

In this study, POS is conceptualized as a multidimensional construct comprising two distinct but related dimensions: affective support and instrumental support [9]. Affective support refers to nurses’ perceptions that the organization cares about their well‐being, values their contributions, and is concerned about their job satisfaction and personal development. Instrumental support, by contrast, refers to nurses’ perceptions that the organization provides the necessary resources, equipment, information, and working conditions to enable them to perform their jobs effectively. While both dimensions contribute to nurses’ overall sense of organizational support, they may operate through somewhat different mechanisms in shaping nurses’ work attitudes and behaviors.

In the nursing context, this theoretical framework suggests that nurses who feel supported by their organization will be motivated to “give back” through behaviors and attitudes that benefit the organization. Professional values—the internalized behavioral norms that guide nursing practice—represent one important avenue for such reciprocation. When nurses feel valued and supported, they are more likely to internalize and enact the professional values that align with high‐quality patient care, thereby fulfilling their reciprocal obligation to the organization. Thus, this study proposes Hypothesis 1: Nurses’ POS can significantly and positively predict professional values.

Nurses’ perceived professional benefits are the harvest and benefits that nurses perceive in their professional careers, and nurses’ recognition of their profession can promote their comprehensive personal growth [10]. The concept was introduced to examine the positive experience of nurses and formulate effective strategies to address the “brain drain” of nurses. Studies have shown that POS can positively predict perceived professional benefits [11, 12]. According to the principle of reciprocity, nurses who feel a high level of POS will develop a sense of reciprocity, thereby maintaining a high level of investment and vitality in their nursing work, continuously learning new knowledge and skills to cope with the pressures and challenges of work. This will enhance the sense of belonging among nurses and make them perceive more benefits from their profession, increasing perceived professional benefits. Research indicates that increasing nurses’ perceived professional benefits can increase their level of professional identification, and a strong sense of professional identification will play a positive incentive role in the development of nurses [13], which is conducive to the cultivation of nurses’ professional values.

The mediating role of perceived professional benefits aligns with the social exchange framework underlying OST. Consistent with [7] comprehensive review, this pattern reflects the norm of reciprocity: nurses who perceive strong organizational support experience greater professional fulfillment—including personal growth, team belonging, and positive professional recognition—and as a form of reciprocal exchange, develop stronger commitment to the professional values that guide excellence in nursing practice. Thus, this study proposes Hypothesis 2: Nurses’ perceived professional benefits mediate the relationship between POS and professional values.

The above research mainly adopts a variable‐centered perspective, aiming to explore which factors affect the formation of nurses’ professional values and their mechanisms of action. While variable‐centered approaches have established important relationships between POS, perceived professional benefits, and professional values, they operate under the assumption that these relationships are uniform across all nurses. However, this assumption may obscure meaningful individual differences in how nurses perceive and respond to organizational support and professional benefits. Nurses are not homogeneous; they vary in personal characteristics, work experiences, and psychological dispositions that may shape distinct patterns of perceived support and benefits.

Latent profile analysis (LPA), an individual‐centered analytical technique, addresses this limitation by identifying heterogeneous subgroups within a population based on similar response patterns across multiple indicators. Unlike variable‐centered methods that focus on relationships between variables, LPA focuses on similarities and differences among individuals, allowing researchers to uncover “hidden” groups that may have different profiles of POS and professional benefits. LPA has been increasingly applied in nursing research to identify heterogeneity in psychological distress, voice behavior, and other outcomes, demonstrating its utility in revealing meaningful subgroup differences that inform targeted interventions.

Identifying such subgroups has important theoretical and practical implications. Theoretically, it advances our understanding of how organizational and individual factors interact to produce distinct patterns of workplace perceptions. Practically, it enables nursing managers to move beyond “one‐size‐fits‐all” interventions and develop targeted strategies tailored to the specific needs of different nurse subgroups. For example, nurses with extremely low support and low professional benefits may require intensive organizational interventions focused on basic support and recognition, while those with moderate support but moderate benefits might benefit from professional development opportunities that enhance their sense of professional fulfillment. Understanding these distinct profiles allows for more efficient allocation of limited organizational resources and more personalized approaches to fostering positive professional values.

Currently, no researchers have used LPA to study the factors influencing nurses’ professional values by examining the combined patterns of POS and perceived professional benefits. Thus, this study attempts to propose Hypothesis 3: There are different combinations of POS‐perceived professional benefits, and there are differences in professional value levels among different subgroups of individuals.

In short, this study aims to use both variable‐centered and individual‐centered analytical approaches to profoundly investigate the relationship and mechanisms between nurses’ POS, perceived professional benefits, and professional values and further explore the heterogeneity of professional values in different categories of POS‐perceived professional benefits. By integrating these complementary approaches, this study aims to provide a more comprehensive understanding of the factors associated with nurses’ professional values and offer evidence‐based guidance for targeted interventions, thereby providing preliminary empirical support for targeted cultivation of positive professional values among nurses and improving the quality of nursing services.

## 2. Materials and Methods

### 2.1. Study Design and Participants

This study employed a cross‐sectional survey design. A convenience sampling method was used to recruit clinical nurses from five hospitals in Heilongjiang Province between September and November 2024. This approach was chosen due to its practicality in accessing the target population within the designated timeframe, a sampling strategy commonly employed in exploratory clinical research where random sampling is logistically challenging. Eligibility criteria included (1) holding a Chinese registered nurse license, (2) having worked at the participating hospital for at least 1 year, and (3) providing voluntary informed consent. The primary analytic method was LPA, which prior research indicates requires a minimum sample size of 300–500 participants [14, 15]. A total of 742 nurses were ultimately recruited and participated in the study.

Participants ranged in age from 20 to 55 years (*M* = 33.04, SD = 7.43). The sample comprised 156 males (21.0%) and 586 females (79.0%). Regarding professional rank, 503 participants (67.8%) held junior titles (nurse or senior nurse), 161 (21.7%) held intermediate titles (supervisor nurse), and 78 (10.5%) held senior titles (associate chief nurse or chief nurse). In terms of work experience, 102 participants (13.7%) had 1–2 years, 136 (18.3%) had 3–5 years, 218 (29.4%) had 6–10 years, 197 (26.5%) had 11–20 years, and 89 (12.0%) had 21 years or more. Regarding monthly income, 350 participants (47.2%) earned < 3000 RMB, 186 (25.1%) earned 3000–5000 RMB, 194 (26.1%) earned 5000–10,000 RMB, and 12 (1.6%) earned > 10,000 RMB.

### 2.2. Measures

#### 2.2.1. POS Scale

The POS scale, revised by Zuo and Yang, was used to measure nurses’ POS [9]. The scale comprises 13 items across two dimensions: affective support and instrumental support. Each item was rated from 1 (significantly unmet) to 5 (significantly met), with higher scores indicating a greater perceived level of support from the organization. The scale has demonstrated reliability and validity when applied to nursing groups [9]. In this study, Cronbach’s α was 0.990 for the full scale and 0.990 and 0.972 for the affective and instrumental subscales, respectively.

#### 2.2.2. Nurses’ Perceived Professional Benefit Questionnaire (NPPBQ)

The simplified version of the NPPBQ [16] was used to assess nurses’ perceived professional benefits. The 17‐item scale includes five dimensions: positive professional perception, good nurse‐patient relationships, recognition from family and friends, team belonging, and personal growth. Responses are recorded on a 5‐point Likert scale from 1 (strongly disagree) to 5 (strongly agree), with higher scores reflecting greater perceived benefits from the nursing profession. The simplified questionnaire has shown good reliability and validity [17]. In the current sample, Cronbach’s α was 0.974 for the full scale and ranged from 0.820 to 0.945 across the five subscales.

#### 2.2.3. Nurse Positive Professional Values Scale (NPVS)

The Chinese version of the NPVS [18] was used to measure nurses’ professional values. The scale consists of 26 items across four dimensions: care provision, activism, trust, responsibility, freedom, and security. Items are rated on a 5‐point Likert scale from 1 (not important) to 5 (very important), with higher scores indicating more positive professional values. The scale is widely used in research on nurses’ professional values [19]. In this study, Cronbach’s α was 0.991 for the full scale and ranged from 0.942 to 0.978 across the four subscales.

### 2.3. Data Analysis

Statistical analyses were performed using SPSS 27.0 and Mplus 8.3. SPSS was used for data management, descriptive statistics, reliability analysis, correlation analysis, and analysis of variance. To test the hypothesized mediation model, we employed Hayes’ SPSS macro PROCESS (Model 4) for mediation analysis with bootstrapping. To account for potential confounding effects, several demographic characteristics were included as covariates in the model, including sex, age, clinical ladder, monthly income, and years of work experience. It should be noted that while this analysis examines the indirect effect consistent with the theoretical model, the cross‐sectional nature of the data precludes definitive causal inferences regarding mediation.

LPA was conducted in Mplus 8.3 to identify subgroups of nurses based on their scores on POS and perceived professional benefits. The optimal number of profiles was determined by evaluating the following model fit indices: Akaike Information Criterion (AIC), Bayesian Information Criterion (BIC), adjusted BIC (aBIC), Lo–Mendell–Rubin likelihood ratio test (LMRT), bootstrapped likelihood ratio test (BLRT), and entropy. Lower values of AIC, BIC, and aBIC indicate better model fits. Significant *p* values for the LMRT and BLRT suggest that a model with *k* profiles provides a significantly better fit than a model with *k* − 1 profiles. Entropy values range from 0 to 1, with values above 0.80 generally indicating adequate classification accuracy; higher entropy reflects greater classification precision [20]. Additionally, the average latent class probabilities were examined, with diagonal probabilities above 0.8 considered acceptable [21]. Finally, analysis of variance was used to examine differences in professional values scores across the identified profile groups.

## 3. Results

### 3.1. Common Method Bias Test

In this study, some scale items were reversely scored to control the mutual influence between scales; at the same time, Harman’s single‐factor test was conducted to assess common method bias. The results showed that the first factor accounted for 36.86% of the total variance, which is below the recommended threshold of 40%. Therefore, common method bias is unlikely to be a serious concern in this study.

### 3.2. Descriptive Statistics of Variables

Table 1 lists each variable’s mean, standard deviation, and correlation matrix. Correlation analysis showed that the three variables of POS, perceived professional benefits, and professional values were positively correlated with each other (0.661∼0.740).

**TABLE 1:** Descriptive statistics of variables.

Variables	*M*	SD	1	2	3
1 Perceived Organizational Support	3.738	0.981	1		
2 Perceived Professional Benefits	4.000	0.772	0.667^∗∗∗^	1	
3 Professional Values	3.802	0.902	0.661^∗∗∗^	0.740^∗∗∗^	1

^∗∗∗^Correlation is significant at the 0.01 level (two‐tailed).

### 3.3. Discriminant Validity

To examine whether the three key constructs—POS, perceived professional benefits (NPPB), and professional values (NPVS)—were empirically distinct, we conducted a series of confirmatory factor analyses (CFAs) using Mplus 8.3. Given the multidimensional nature of the scales, we used dimension parceling as indicators for each latent construct. Specifically, POS was indicated by its two dimensions, NPPB by its five dimensions, and NPVS by its four dimensions.

We compared the fit of the hypothesized three‐factor model against several alternative models, including three two‐factor models (combining pairs of constructs) and a one‐factor model (combining all constructs). As shown in Supporting Table S1, the three‐factor model demonstrated a good fit to the data (*χ* ^2^/df = 5.76, CFI = 0.97, TLI = 0.95, RMSEA = 0.08, SRMR = 0.02) and fit significantly better than all alternative models (*p* < 0.001). These results provide empirical evidence for the discriminant validity of the three measures.

### 3.4. The Relationship Model of POS, Perceived Professional Benefits, and Professional Values—Variable‐Centered Analysis

Using Hayes’ macro PROCESS (Model 4) for SPSS, a mediation analysis was conducted to examine the indirect effect of POS on professional values through perceived professional benefits, while controlling for demographic variables including sex, age, clinical ladder, monthly income, and years of work experience.

**FIGURE 1:**
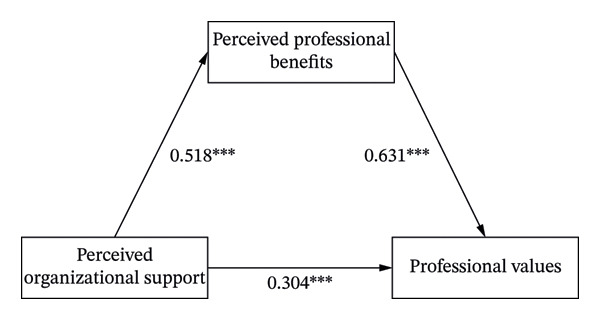
The mediation model diagram of perceived organizational support and nurses’ professional values. Note: ^∗∗∗^Correlation is significant at the 0.001 level (two‐tailed).

The results of the mediation analysis are presented in Figure 1. POS was positively and significantly associated with nurses’ perceived professional benefits (*B* = 0.518, 95% CI [0.476, 0.561], *p* < 0.001). Perceived professional benefits were also significantly positively associated with nurses’ professional values (*B* = 0.631, 95% CI [0.559, 0.704], *p* < 0.001). When perceived professional benefits were included as a mediator, the indirect effect of POS on professional values via perceived professional benefits was significant (indirect effect = 0.327, 95% CI [0.249, 0.414]; see Table 2). In this model, the direct association between POS and nurses’ professional values remained significant (*B* = 0.304, 95% CI [0.222, 0.336], *p* < 0.001). Thus, within the correlational pattern observed in this cross‐sectional data, perceived professional benefits partially mediated the relationship between POS and nurses’ professional values, accounting for 51.8% of the total effect.

**TABLE 2:** Direct and indirect effect statistics table.

Model pathways	Effect	SE	95% CI		Relative mediating effect (%)
			LLCI	ULCI	
Direct effects	0.302	0.029	0.221	0.333	45.60
Indirect effects	0.360	0.046	0.275	0.451	54.40

*Note:* LLCI, ULCI refer to the lower and upper limits of the 95% confidence interval obtained by the bias‐corrected percentile Bootstrap method.

### 3.5. Prediction of Professional Values by POS and Perceived Professional Benefits—Individual‐Centered Analysis

Building on the variable‐centered findings, which demonstrated a significant partial mediating effect of perceived professional benefits, we conducted LPA to further explore differences in professional values across nurse subgroups based on their POS and perceived professional benefits scores.

To identify the optimal class solution, we specified models ranging from two to six profiles. The fit indices are presented in Table 3. Although the six‐profile model showed the lowest AIC, BIC, and aBIC values and the highest entropy, its LMRT was not significant (*p* > 0.05), indicating that the six‐profile model did not significantly improve upon the five‐profile model. In contrast, the five‐profile model demonstrated excellent fit, with AIC, BIC, and aBIC values substantially lower than those of the four‐profile model, an entropy of 0.982, significant LMR and BLRT tests (*p* < 0.05), and average latent class probabilities all exceeding 0.8 (see Table 4). Therefore, the five‐profile model was selected as the optimal solution.

**TABLE 3:** Fit indices of LPA for nurses’ perceived organizational support and perceived professional benefit profiles (*N* = 742).

Profiles	AIC	BIC	aBIC	Entropy	LMR	BLRT	Proportion
1	3806.08	3824.52	3811.82				
2	3551.38	3583.65	3561.42	0.723	0.0092	< 0.001	0.791/0.209
3	3288.63	3334.73	3302.97	0.877	< 0.001	< 0.001	0.087/0.647/0.266
4	2870.85	2930.77	2889.49	0.967	< 0.001	< 0.001	0.535/0.151/0.027/0.287
**5**	**2664.64**	**2738.38**	**2687.58**	**0.982**	**0.0096**	**< 0.001**	**0.049/0.053/0.429/0.245/0.225**
6	2567.60	2655.18	2594.85	0.982	0.2367	< 0.001	0.049/0.053/0.426/0.245/0.221/0.007

*Note:* The bold values indicate the fit indices for the selected optimal model, that is, the five‐profile solution retained in the final analysis.

**TABLE 4:** Average latent class probabilities for the most likely latent members by potential category (column).

Category	Number of subjects (%)	C1	C2	C3	C4	C5
C1	167 (22.5%)	0.997	0.003	0.000	0.000	0.000
C2	318 (42.9%)	0.001	0.964	0.000	0.035	0.000
C3	182 (24.5%)	0.000	0.000	0.988	0.010	0.002
C4	39 (5.3%)	0.000	0.003	0.011	0.987	0.000
C5	36 (4.9%)	0.000	0.000	0.004	0.000	0.996

**FIGURE 2:**
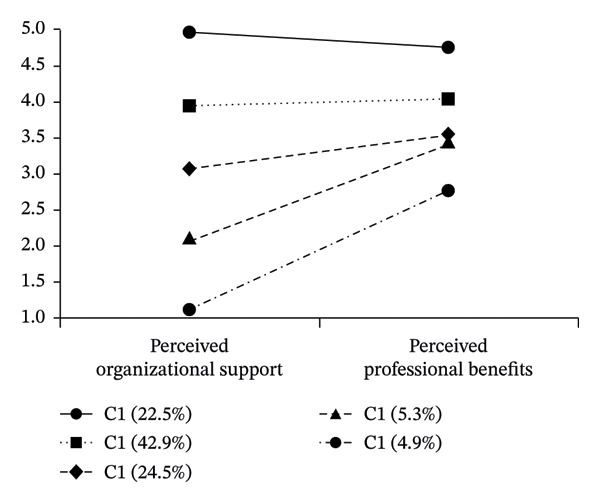
Latent profile analysis results (*N* = 742).

Based on the characteristics of POS and perceived professional benefits, the five profiles were labeled as follows: (1) extremely high support–extremely high benefit group, (2) high support–high benefit group, (3) moderate support–moderate benefit group, (4) low support–moderate benefit group, and (5) extremely low support–low benefit group (see Figure 2).

The distribution of nurses across these five profiles was as follows: the high support–high benefit group was the largest, comprising 318 nurses (42.9%), followed by the moderate support–moderate benefit group with 182 nurses (24.5%), the extremely high support–extremely high benefit group with 167 nurses (22.5%), the low support–moderate benefit group with 39 nurses (5.3%), and the extremely low support–low benefit group with 36 nurses (4.9%).

One‐way analysis of variance revealed significant differences in professional values scores across the five profiles (*F* = 171.282, *p* < 0.001, *η* ^2^ = 0.48). Post hoc comparisons indicated that all pairwise differences were statistically significant (*p* < 0.001) except between the moderate support–moderate benefit group (C3) and the low support–moderate benefit group (C4), which did not differ significantly (see Table 5).

**TABLE 5:** Comparison of nurses’ professional values scores in different perceived organizational support‐perceived professional benefit potential categories.

Category	Professional values (*M* ± SD)	*F*	*η* ^2^	Post‐hoc test (*p* < 0.001)
C1	4.84 ± 0.36	171.282^∗∗∗^	0.48	C1 > C2 > C3/C4 > C5
C2	3.75 ± 0.65			
C3	3.28 ± 0.67			
C4	3.18 ± 0.82			
C5	2.68 ± 1.17			

^∗∗∗^Correlation is significant at the 0.001 level (two‐tailed).

## 4. Discussion

This study adopted both variable‐centered and individual‐centered analytical approaches. First, it examined the correlational link between POS and nurses’ professional values, along with the indirect role of perceived professional benefits within this relationship. Subsequently, it explored the potential latent profiles derived from the combination of POS and perceived professional benefits and compared the professional values scores across different profile groups. These two analytical approaches complement each other and contribute to a more nuanced understanding of the factors associated with nurses’ professional values, which can inform future tailored intervention strategies.

### 4.1. The Relationship Between POS and Nurses’ Professional Values: The Mediating Role of Perceived Professional Benefits

The findings indicate that Hypothesis 1 was supported, as POS demonstrated a significant positive correlation with nurses’ professional values. In other words, nurses who reported higher levels of POS tended to hold more positive professional values. This finding aligns with OST, which posits that employees who perceive greater support from their organization reciprocate through positive attitudes and behaviors that benefit the organization [6, 7]. Professional values—the internalized behavioral norms that guide nursing practice—represent one important manifestation of such reciprocation. When nurses feel valued and supported by their organization, they are more likely to internalize and enact the professional values that align with high‐quality patient care, thereby fulfilling their reciprocal obligation to the organization.

It is noteworthy that the POS scale used in this study distinguishes between two dimensions: affective support (e.g., caring about nurses’ well‐being, valuing their contributions) and instrumental support (e.g., providing adequate resources, equipment, and working conditions). Both dimensions contribute to nurses’ overall sense of organizational support, but they may operate through somewhat different mechanisms. Affective support primarily fulfills nurses’ socioemotional needs, strengthening their emotional bond with the organization and enhancing their sense of belonging and self‐worth. Instrumental support, by contrast, provides the practical resources necessary for nurses to perform their duties effectively, reducing work‐related stress and enabling them to deliver high‐quality care. According to OST, both forms of support signal that the organization values nurses’ contributions and cares about their well‐being, thereby triggering the norm of reciprocity [7]. However, affective support may be particularly important for fostering internalized professional values, as it directly addresses nurses’ psychological needs for esteem, affiliation, and emotional security—needs that are closely linked to value internalization processes.

Furthermore, the results support Hypothesis 2, indicating that perceived professional benefits partially mediated the association between POS and professional values. This finding aligns with prior research suggesting that when nurses feel valued and supported by their organization, they are likely to experience enhanced psychological well‐being, greater job engagement, and increased satisfaction, thereby perceiving their profession as more rewarding [22]. Such positive perceptions of professional benefits may, in turn, contribute to the development of stronger professional values. These observations are consistent with social exchange theory [23], which posits that when nurses receive adequate organizational support and feel fairly rewarded in their roles, they are motivated to reciprocate through higher work engagement and improved service quality. This reciprocal process can strengthen nurses’ professional identity and support the internalization of constructive professional values.

### 4.2. Differences in Professional Values Among Nurses With Different POS‐Perceived Professional Benefit Categories

Building on the variable‐centered analysis, this study further adopted an individual‐centered perspective to identify distinct subgroups of nurses based on their patterns of POS and perceived professional benefits. The results identified five distinct latent profiles: an “extremely high support–extremely high benefit” group (22.5%), a “high support–high benefit” group (42.9%), a “moderate support–moderate benefit” group (24.5%), a “low support–moderate benefit” group (5.3%), and an “extremely low support–low benefit” group (4.9%). These findings support Hypothesis 3 and reveal substantial heterogeneity in how nurses perceive organizational support and professional benefits.

The largest group was the “high support–high benefit” group, comprising 42.9% of the sample, followed by the “moderate support–moderate benefit” group (24.5%) and the “extremely high support–extremely high benefit” group (22.5%). Collectively, these three groups accounted for nearly 90% of the sample, suggesting that most nurses in this study reported moderate to high levels of both POS and perceived professional benefits. This is encouraging, as it indicates that the majority of nurses feel reasonably supported by their organizations and derive meaningful benefits from their profession.

However, two smaller but clinically significant subgroups emerged that warrant particular attention. The “low support–moderate benefit” group (5.3%) reported low organizational support despite moderate professional benefits. This pattern suggests that these nurses derive personal and professional fulfillment from their work—perhaps through patient interactions, collegial relationships, or intrinsic satisfaction—but do not feel adequately supported by their organization. This disconnect may place them at risk for burnout or turnover if organizational support does not improve, as they may eventually question why they should remain committed to an organization that does not reciprocate their investment.

The “extremely low support–low benefit” group (4.9%), while the smallest, represents the most vulnerable subgroup. These nurses report minimal organizational support and perceive few benefits from their profession, placing them at highest risk for professional disengagement, burnout, and turnover intention. The extremely low professional values scores in this group (*M* = 2.68, significantly lower than all other groups) confirm their heightened vulnerability and underscore the urgent need for targeted interventions.

Significant differences in professional values were observed across the five profiles, with professional values scores decreasing progressively from the “extremely high support–extremely high benefit” group to the “extremely low support–low benefit” group. This gradient pattern provides additional evidence for the cumulative effect of POS and perceived professional benefits on professional values. Notably, while the “low support–moderate benefit” group scored lower than the “moderate support–moderate benefit” group, this difference did not reach statistical significance, suggesting that moderate levels of professional benefits may partially buffer the negative effects of low organizational support on professional values. This finding aligns with the conservation of resources theory, which posits that individuals with greater resources are better equipped to cope with resource loss. In this context, nurses with moderate professional benefits may have sufficient psychological resources to maintain relatively positive professional values despite low organizational support.

These findings have important implications for nursing management. First, the identification of distinct profiles underscores the need for differentiated, targeted interventions rather than uniform approaches. Nursing managers should assess nurses’ levels of both POS and perceived professional benefits to identify which subgroup they belong to and tailor interventions accordingly. Second, the vulnerable subgroups—particularly the “extremely low support–low benefit” group—require immediate attention. For these nurses, interventions should focus on addressing basic support needs, including ensuring safe working conditions, providing emotional support, and demonstrating genuine care for their well‐being. Third, even among nurses in the moderate and high groups, continuous efforts are needed to sustain and enhance positive perceptions. This can include providing ongoing professional development opportunities, fostering positive team dynamics, and regularly recognizing nurses’ contributions.

From a theoretical perspective, these findings extend OST by demonstrating that POS and perceived professional benefits combine in complex ways to produce distinct psychological profiles. The heterogeneity observed in this study suggests that the relationship between organizational support and professional outcomes is not uniform but varies across individuals based on their unique configurations of workplace perceptions. Future research should examine whether these profiles predict different long‐term outcomes, such as turnover, burnout, and patient care quality, and whether they are stable or change over time in response to organizational interventions.

### 4.3. Implications

These findings have important theoretical implications. By integrating OST with social exchange theory, this study provides a theoretical framework for understanding how organizational factors shape nurses’ professional values through the mediating mechanism of perceived professional benefits, extending previous research that has primarily focused on individual and demographic determinants. The distinction between affective and instrumental support reveals that different dimensions of organizational support may have differential effects on nurses’ work attitudes and values, advancing OST by demonstrating that different forms of support may trigger reciprocal processes through distinct psychological mechanisms. Furthermore, the integration of variable‐centered and individual‐centered approaches reveals heterogeneity in how POS and perceived professional benefits combine to influence professional values, showing that the relationship between organizational support and professional outcomes varies across individuals based on their unique configurations of workplace perceptions.

These findings also have important practical implications for nursing management. To cultivate positive professional values, nursing managers should enhance both affective and instrumental organizational support through strategies grounded in OST. For affective support, this includes conducting regular one‐on‐one meetings to listen to nurses’ concerns and acknowledge their contributions, implementing formal recognition programs such as “Nurse of the Month” awards, involving nurses in decision‐making processes, demonstrating genuine care by checking in during stressful periods and celebrating milestones, and providing mentorship and professional development support. For instrumental support, organizations should maintain adequate staffing levels and appropriate nurse‐to‐patient ratios, ensure access to modern equipment and safe working conditions, offer fair compensation and performance‐based incentives, provide continuing education opportunities, and allocate adequate time and resources for task completion. Additionally, given that perceived professional benefits mediate this relationship, interventions should also target nurses’ professional benefits through structured mentorship programs, team‐building activities that foster belonging, regular constructive feedback highlighting achievements, sharing positive patient outcomes, and creating clear career advancement pathways. Implementing these theory‐grounded strategies can create a supportive organizational environment that fosters positive professional values, ultimately contributing to improved patient care quality, reduced turnover, and a more resilient nursing workforce.

## 5. Limitations and Future Directions

This study has several limitations that should be acknowledged when interpreting the findings. First, the cross‐sectional design precludes causal inferences regarding the relationships among POS, perceived professional benefits, and professional values. Future research should employ longitudinal designs to examine how these relationships unfold over time and to test the directionality of the mediated effects.

Second, all data were collected through self‐report questionnaires, which may introduce common method bias. Future research could incorporate multiple data sources, such as supervisor ratings of nurses’ professional behavior, patient satisfaction scores, or objective indicators of turnover and retention, to provide a more comprehensive assessment.

Third, the generalizability of our findings may be limited by the sampling method and geographic scope. Future research should replicate this study in diverse geographic regions and healthcare settings to examine the robustness and generalizability of the findings.

Finally, this study focused on POS and perceived professional benefits as predictors of professional values. However, professional values are likely influenced by multiple factors at the individual, interpersonal, organizational, and societal levels. Future research should adopt a more comprehensive ecological framework that examines how factors at different levels—such as individual characteristics, team dynamics, organizational policies, and societal attitudes toward nursing—interact to shape nurses’ professional values over time.

Despite these limitations, this study makes important contributions by demonstrating both the mediating role of perceived professional benefits in the POS‐professional values relationship and the heterogeneity in how POS and perceived professional benefits combine to influence professional values. By integrating variable‐centered and individual‐centered approaches, this study provides a more nuanced understanding of the factors associated with nurses’ professional values and offers evidence‐based guidance for targeted interventions. Future research should build on these findings using longitudinal designs, diverse samples, and multimethod assessments to further advance our understanding of how to cultivate and sustain positive professional values among nurses.

## 6. Conclusions

This study employed both variable‐centered and individual‐centered approaches to examine the relationships among POS, perceived professional benefits, and professional values in nurses. The findings revealed that perceived professional benefits partially mediated the relationship between POS and professional values. Furthermore, five distinct profiles of POS and perceived professional benefits were identified, which demonstrated significant differences in professional values. These findings advance the understanding of how organizational and individual factors jointly shape nurses’ professional values and provide evidence‐based guidance for developing targeted interventions to foster positive professional values among nurses.

## Funding

This work was supported by the Humanities and Social Sciences Project, Ministry of Education (grant number 24YJAZH005), China; and the Natural Science Foundation of Heilongjiang Province (grant number LH2024H031), China.

## Ethics Statement

The study was approved by the Ethics Committee of the Daqing Campus of Harbin Medical University (approval number: HMUDQ20241224001). Participants were informed about the purpose of the study, and their consent was obtained. To ensure the participants’ privacy, all data were available only to research team members.

## Conflicts of Interest

The authors declare no conflicts of interest.

## Supporting Information

Supporting Table S1 presents the confirmatory factor analysis results testing the discriminant validity of perceived organizational support (POS), perceived professional benefits (NPPB), and professional values (NPVS). The three‐factor model demonstrated good fit and significantly outperformed all alternative models, supporting the discriminant validity of the three measures.

## Supporting information


**Supporting Information** Additional supporting information can be found online in the Supporting Information section.

## Data Availability

The data that support the findings of this study are available on request from the corresponding author. The data are not publicly available due to privacy or ethical restrictions.
