# Supplementary material for: The Relationship Between Perceived Organizational Support, Perceived Professional Benefits, and Professional Values Among Nurses: Variable‐Centered and Individual‐Centered Analyses
Source: J Nurs Manag. 2026 Apr 29;2026:9608844. doi: 10.1155/jonm/9608844 (PMC13126084; doi:10.1155/jonm/9608844)
Supplement: Supplementary file 1 — Supporting Information Additional supporting information can be found online in the Supporting Information section. [file JONM-2026-9608844-s001.docx]

| Model | Fit Indices | | | | | | Model Comparison | |
| --- | --- | --- | --- | --- | --- | --- | --- | --- |
|  | χ^2^/df | CFI | TLI | RMSEA | SRMR | ΔCFI | | ΔRMSEA |
| Three-factor model | 5.76 | 0.97 | 0.95 | 0.08 | 0.02 | — | | — |
| Two-factor model (POS+NPPB combined) | 18.28 | 0.88 | 0.84 | 0.15 | 0.07 | 0.09^a^ | | 0.07^a^ |
| Two-factor model (POS+NPVS combined) | 17.40 | 0.88 | 0.84 | 0.15 | 0.08 | 0.09^a^ | | 0.07^a^ |
| Two-factor model (NPPB+NPVS combined) | 39.05 | 0.71 | 0.63 | 0.23 | 0.14 | 0.26^a^ | | 0.15^a^ |
| One-factor model | 47.52 | 0.64 | 0.55 | 0.25 | 0.14 | 0.33^a^ | | 0.17^a^ |

Table S1 Measurement Invariance

*Note*: *a* Comparison with the three-factor model. *POS* represents Perceived Organizational Support. *NPPB* represents Perceived Professional Benefits. *NPVS* represents Professional Values.
